# Case Report: Longitudinal monitoring of clonal evolution by circulating tumor DNA for resistance to anti-EGFR antibody in a case of metastatic colorectal cancer

**DOI:** 10.3389/fonc.2023.1203296

**Published:** 2023-06-26

**Authors:** Tamotsu Sagawa, Yasushi Sato, Masahiro Hirakawa, Kyoko Hamaguchi, Fumito Tamura, Hiroyuki Nagashima, Koshi Fujikawa, Koichi Okamoto, Yutaka Kawano, Masahiro Sogabe, Hiroshi Miyamoto, Tetsuji Takayama

**Affiliations:** ^1^ Department of Gastroenterology, Hokkaido Cancer Center, Sapporo, Hokkaido, Japan; ^2^ Department of Gastroenterology and Oncology, Tokushima University Graduate School of Biomedical Sciences, Tokushima, Japan

**Keywords:** anti-EGFR antibody, clonal evolution, ctDNA, metastatic colorectal cancer, resistance

## Abstract

**Background:**

Treatment with anti-EGFR antibody has been shown to prolong survival in patients with RAS wild-type metastatic colorectal cancer (mCRC). However, even patients who initially respond to anti-EGFR antibody therapy, almost without exception, develop resistance to the therapy and then fail to respond. Secondary mutations in the mitogen-activated protein (MAPK) signaling pathway (mainly in NRAS and BRAF) have been implicated in anti-EGFR resistance. However, the process by which resistant clones develop during therapy has not been elucidated, and considerable intrapatient and interpatient heterogeneity exists. Circulating tumor DNA (ctDNA) testing has recently allowed the noninvasive detection of heterogeneous molecular alterations that underlie the evolution of resistance to anti-EGFR. In this report, we describe our observation of genomic alterations in *KRAS* and *NRAS* in a patient with acquired resistance to anti-EGFR antibody drugs by tracking clonal evolution using serial ctDNA anaylsis.

**Case presentation:**

A 54-year-old woman was initially diagnosed with sigmoid colon cancer with multiple liver metastases. After receiving first-line mFOLFOX + cetuximab, second-line FOLFIRI + ramucirumab, third-line trifluridine/tipiracil + bevacizumab, fourth-line regorafenib, and fifth-line CAPOX + bevacizumab, she was rechallenged with CPT-11 + cetuximab. The best response to anti-EGFR rechallenge therapy was a partial response. *RAS* in the ctDNA was assessed during treatment. The *RAS* status changed from wild type to mutant type, back to wild type, and again to mutant type (*NRAS/KRAS* codon 61) during the course of treatment.

**Conclusion:**

In this report, tracking of ctDNA allowed us to describe clonal evolution in a case in which we observed genomic alterations in *KRAS* and *NRAS* in a patient who acquired resistance to anti-EGFR antibody drugs during treatment. It is reasonable to consider repeat molecular interrogation during progression in patients with mCRC by using ctDNA analysis, which could help to identify patients who may benefit from a rechallenge strategy.

## 1 Introduction

Colorectal cancer (CRC) is the third most common malignancy and the second most deadly cancer worldwide, with an estimated 1.9 million cases and 0.9 million deaths worldwide in 2020 (1). In the past few years, advances in tumor biology, molecular genetics, and the introduction of molecularly targeted drugs have revolutionized the treatment of patients with metastatic CRC (mCRC).

Epidermal growth factor receptor (EGFR) is an important target for CRC treatment, and the combination of anti-EGFR monoclonal antibodies (mAbs) (cetuximab and panitumumab) and cytotoxic chemotherapy has become the standard treatment for patients with *RAS* wild-type mCRC, given its clinical efficacy and the extended survival it achieves (2, 3). In contrast, *RAS* mutations are negative predictors of anti-EGFR mAb efficacy and serve as primary and secondary resistance markers (4).

RAS is a family of small GTPases that act as a molecular switch in the pathway. In its active state, RAS-GTP interacts with downstream effectors, such as RAF kinases, leading to activation of the MAPK/ERK and PI3K/AKT signaling pathways. These pathways promote cell growth, survival, and proliferation (5). However, RAS mutations disrupt the normal regulation of the EGFR-RAS pathway, leading to constitutive activation of RAS and bypassing its dependency on EGFR signaling. This mechanism of resistance to anti-EGFR therapies highlights the importance of identifying RAS mutation status in patients before planning treatment for CRC (2, 3). The *RAS* oncogene family includes *KRAS*, *NRAS*, and *HRAS*. CRC-associated mutations occur most commonly in *KRAS*, with approximately 40% of CRC cases harboring *KRAS* mutations, while *NRAS* and *HRAS* mutations are rarely detected in CRC cases (5).

Even in patients with wild-type *RAS*, the emergence of resistant tumor cell populations is inevitable, leading to treatment failure (6). The emergence of treatment resistance is due to the spatial and temporal molecular heterogeneity of tumors caused by the evolution of cancers in adaptation to therapeutic perturbations (7). Thus, selection pressure by anti-EGFR drugs is considered to be one of the most consistent causes of resistance, stimulating an increase in initially silent resistant clones, which attenuate in a time-dependent manner after discontinuation of the anti-EGFR drugs (8). This provides a rationale for the possibility of rechallenge with anti-EGFR therapy in later lines of treatment.

Santini et al. first demonstrated that rechallenging patients with cetuximab could have clinical benefits in mCRC patients, and reported promising results, with a response rate (RR) of 53.8% and a median progression-free survival (PFS) of 6.6 months (9).

For late-line treatment of mCRC, trifluridine/tipiracil (FTD/TPI) + bevacizumab showed promising outcomes, with a median PFS of 5.6 months and a RR of 6.3% (10). However, further treatment options in the late line are desired from the perspective of treatment strategy in the continuum of care. Therefore, rechallenge with anti-EGFR mAbs is a promising therapeutic strategy that is expected to yield high response rates.

However, there are several limitations when considering rechallenge with anti-EGFR mAbs, including the need for biopsy tissue collection to assess *RAS* alterations, potential complications, invasiveness of the procedure, difficulty in tissue collection, and tumor heterogeneity.

Currently, the presence or absence of *RAS* mutations can be confirmed multiple times using using *RAS*-specific circulating tumor DNA (ctDNA) analyses of liquid biopsies. Commercially available kits include the OncoBEAM™ RAS CRC kit and the Comprehensive Cancer Genome Profiling Test (CGP), such as the Foundation One^®^ Liquid CDx. The CRICKET trial, a single-arm Phase II study, demonstrated the efficacy of rechallenge with anti-EGFR antibody therapy (11). In this study, liquid biopsy was used retrospectively to verify the *RAS* and *BRAF* status at the start of the rechallenge. Patients were eligible for the study if they were *RAS* and *BRAF* wild-type mCRC patients who had achieved at least a partial response (PR) and a PFS of at least 6 months on cetuximab- and CPT-11-based primary therapy and who subsequently became resistant. The objective RR was 21%. Of note, patients with wild-type *RAS* showed significantly improved PFS (median PFS 4.0 months vs. 1.9 months, hazard ratio 0.44, 95% confidence interval: 0.18–0.98, p = 0.03) as compared to patients with ctDNA *RAS* mutations, and the overall survival and RR were similarly favorable.

Moreover, in the CHRONOS study, the first open-label, single-arm Phase II trial to evaluate the efficacy of EGFR inhibitor rechallenge prospectively based on ctDNA mutation status, among patients with no detectable changes in ctDNA *RAS*, *BRAF*, or *EGFR* extracellular domain (ECD), eight patients (30%) achieved PR with anti-EGFR rechallenge therapy with panitumumab. These clinical results demonstrated that patient selection based on ctDNA can better select appropriate candidates for anti-EGFR mAb rechallenge (12). In addition, the CITRIC trial is an ongoing study comparing the efficacy of cetuximab + CPT-11 rechallenge in third-line therapy with that of the physicians’ choice of therapy in selected patients with *RAS*, *BRAF*, and *EGFR*-ECD wild-type mCRC, using next-generation sequencing panels (13).

These results will establish the utility of serial ctDNA to guide anti-EGFR rechallenge, which is expected to become an important treatment strategy in the continuum of care for patients with mCRC. However, there are still unresolved issues, such as the clinical factors, length of anti-EGFR-free interval, and therapy that should precede anti-EGFR therapy. Knowing this would help to identify patients who would benefit from a rechallenge strategy. Moreover, it is not clear whether a liquid biopsy-based genetic profile, based on plasma *RAS* mutations alone, is sufficient for patient selection (14). Therefore, further studies are needed to verify that liquid biopsy can be used appropriately in clinical practice.

We here report a case in which we observed genomic alterations in *KRAS* and *NRAS* in a patient with acquired resistance to anti-EGFR antibody drugs, by tracking clonal evolution during the course of treatment using repeated ctDNA analysis, which allowed successful rechallenge with anti-EGFR mAb therapy.

## 2 Case presentation

A 54-year-old woman was initially diagnosed with sigmoid colon cancer with multiple liver metastases. Pathological findings revealed a well-differentiated adenocarcinoma.

**Figure 1:**
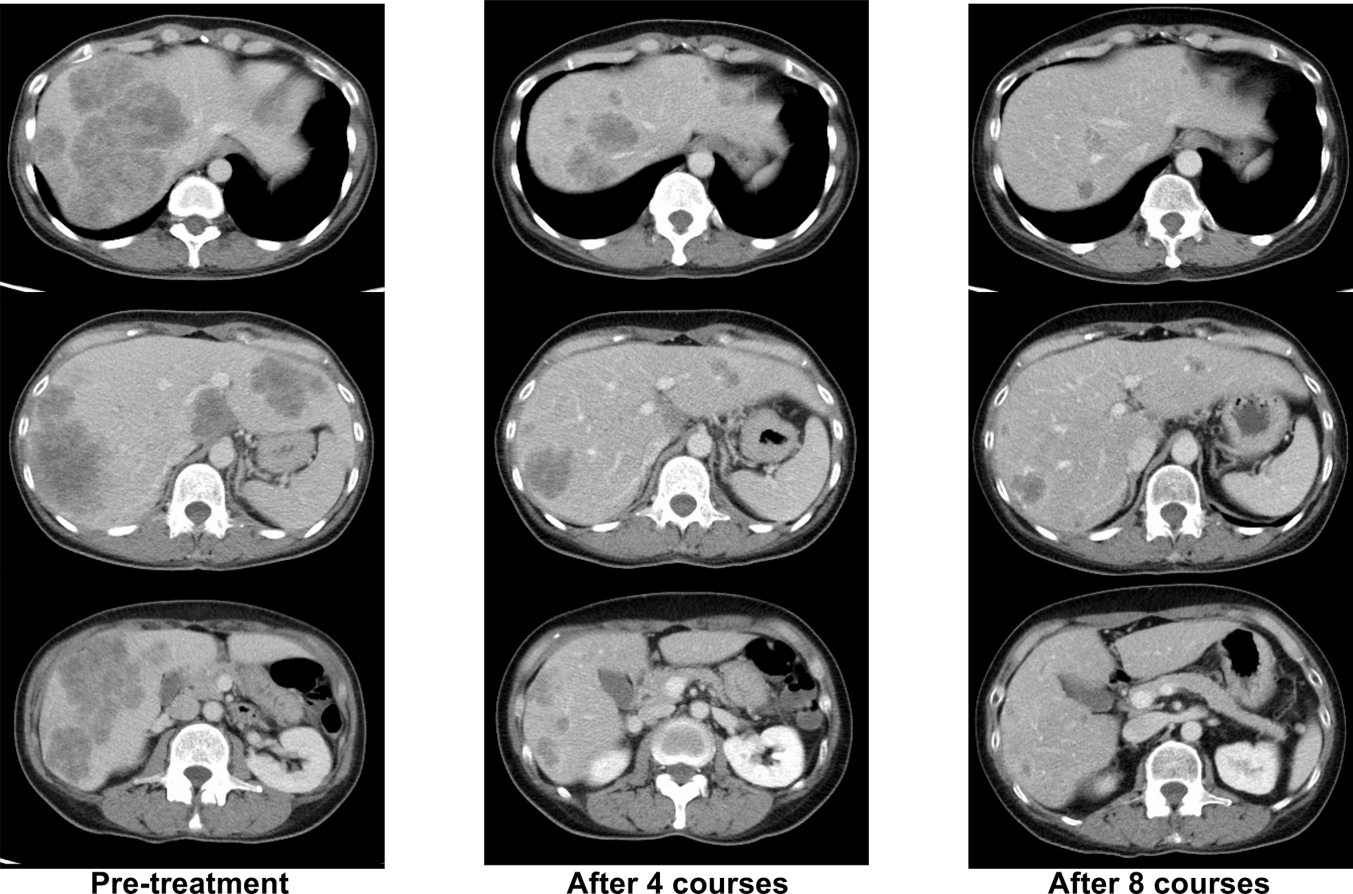
Representative computed tomography (CT) images of liver lesions during ^-^firstline mFOLFOX6+cetuximab treatment.

Her *RAS*/*BRAF* status from the primary tumor were wild-type as determined by using the MEBGEN RASKET™-B kit (Medical and Biological Laboratories Co., Ltd., Nagoya, Japan), which can simultaneously examine 12 types of *RAS* exon 2 (G12S, G12C, G12R, G12D, G12 V, G12A, G13S, G13C, G13R, G13D, G13V, and G13A), 8 types of *RAS* exon 3 (A59T, A59G, Q61K, Q61E, Q61L, Q61P, Q61R, and Q61H), 4 types of *RAS* exon 4 (K117N, A146T, A146P, and A146V) mutations, and *BRAF* exon 15 (V600E). Her tumor status was microsatellite stability (MSS), HER2-negative, and tumor mutational burden–low. Therefore, mFOLFOX6 + cetuximab was started as primary therapy. A deep and long-lasting PR, with a response duration of 16 months, was obtained (**Figure 1**).

Second-line treatment with FOLFIRI + ramucirumab was initiated, after which the patient remained free of disease progression for 6 months. Then, imaging studies revealed progressive disease (PD), as evidenced by metastatic lesions in the liver.

Before the third-line treatment, we examined the presence of *RAS* mutations in consideration of possible anti-EGFR rechallenge. We used the OncoBEAM™ CRC kit (Sysmex Inostics, Hamburg, Germany), which detects 34 mutations in *KRAS/NRAS* codons 12, 13, 59, 61, 117, and 146 in plasma (mutated amino acids cannot be measured using this method, except for G13D at *KRAS* codon 13 and A146T at *NRAS* codon 146), using the cut-off defined as the number of beads with amplified-mutant molecules specifically set per codon. Liquid biopsy revealed mutations in *KRAS* exon 2 (codons 12, 13), *KRAS* exon 4B (codon 146), *NRAS* exon 2 (codons 12, 13), *NRAS* exon 3 (codon 61), and *NRAS* exon 4A (codon 117) (**Supplementary Table 1**).

Subsequently, FTD/TPI+bevacizumab was administered as the third-line treatment, regorafenib as the fourth-line treatment, and CAPOX+ bevacizumab as the fifth-line treatment.

**Figure 2:**
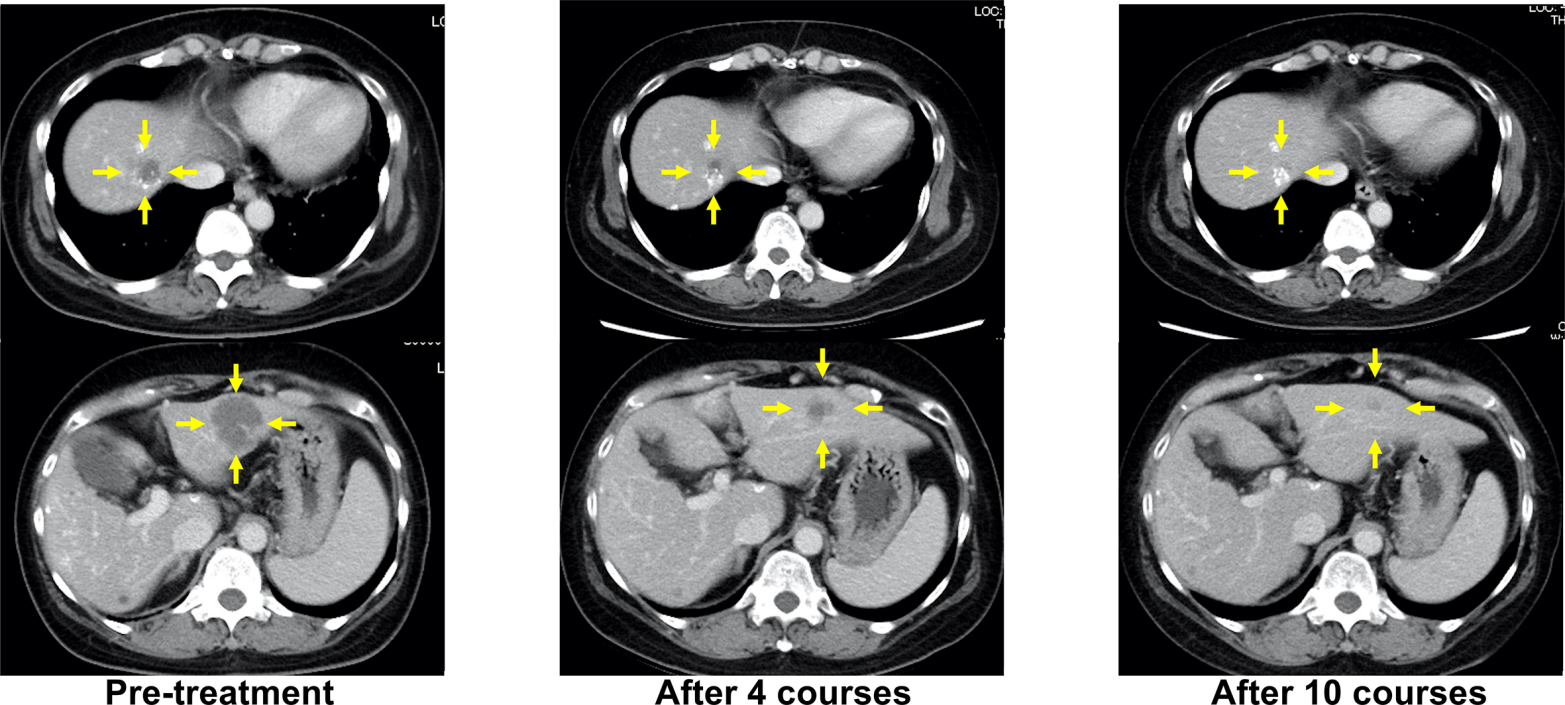
Representative computed tomography (CT) images of liver lesions during anti-EGFR antibody rechallenge treatment (sixth-line, CPT-11+cetuximab). Yellow arrows indicate liver metastases.

When progressive disease (PD) developed after the fifth treatment, her *RAS* status was again tested using the OncoBEAM™ CRC kit. The second analysis of liquid biopsy results revealed no mutations in either *KRAS* or *NRAS*. CPT-11 + cetuximab was administered as the sixth-line treatment, as an anti-EGFR antibody rechallenge therapy. The best response to anti-EGFR rechallenge therapy was PR (**Figure 2**). The duration of response to CPT-11 + cetuximab therapy was 8 months. Subsequently, liver metastases recurred, and PD was confirmed.

Therefore, to search for genetic alterations associated with available targeted therapies, FoundationOne^®^ Liquid CDx (Foundation Medicine, Cambridge, MA), which involves next generation sequencing-based analysis that provides comprehensive genomic profiling to detect a wide range of genetic alterations in 324 genes, utilizing ctDNA, was used. *KRAS* exon 3 (codon 61H) and *NRAS* exon 3 (codon 61L) were identified as actionable mutations. Of note, the *KRAS* codon 61H mutation was different from those previously identified using the OncoBEAM CRC kit (**Supplementary Table 1**).

**Figure 3:**
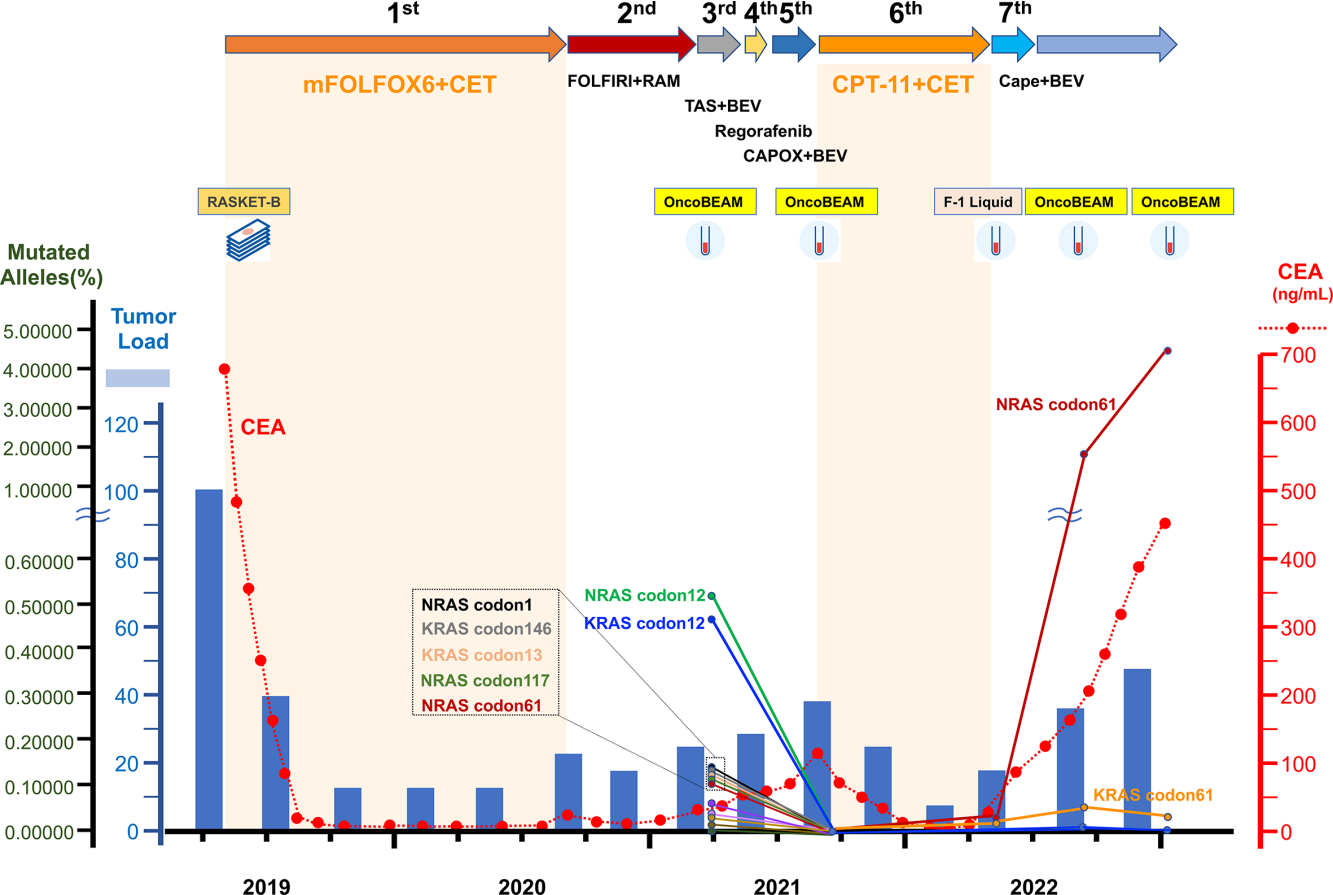
Schematic representation of the dynamics of RAS-altered clones monitored through circulating tumor DNA from the patient. Each treatment received by the patient is indicated on the graph. Blue bars represent variation of tumor load, compared to baseline, during treatments as specified above the graphs. Tumor load was calculated as percentage change based on measurable disease at initiation of treatment (baseline), set as 100%. The dotted red line indicates the changes in carcinoembryonic antigen (CEA) values (ng/ml). The solid lines show the course of the frequency of each RAS mutation (percentage of alleles) detected in circulating DNA at the indicated time points. Each RAS mutation is indicated by a differently colored solid line. CET, cetuximab; RAM, ramucirumab; TAS, tipiracil hydrochloride; BEV, bevacizumab; CAPOX, capecitabine and oxaliplatin; Cape,capecitabine; FOLFIRI, folinic acid, 5-fluorouracil and CPT-11; mFOLFOX, folinic acid, fluorouracil and oxaliplatin; F-1 Liquid, FoundationOne^®^ Liquid CDx.

It has been reported that *RAS*-mutated clones that emerge in anti-EGFR-resistant tumors begin to decay after discontinuation of anti-EGFR therapy, with a half-life of 4.3 months (8). Therefore, to investigate the possibility of a second attempt with anti-EGFR rechallenge therapy, intervening treatment lines without anti-EGFR therapy, we used OncoBEAM to check her ctDNA *RAS* status the third time, 4 months after CPT-11 + cetuximab administration, and the fourth time, 7 months after CPT-11 + cetuximab administration. The results showed a rapid increase in the *NRAS* codon 61 mutation, and although capecitabine + bevacizumab was administered as the seventh-line treatment, tumor progression and a rapid increase in carcinoembryonic antigen (CEA) were observed, leading to receive subsequent best supportive care for this patient. A schematic representation of the patient’s clinical history is shown in **Figure 3**.

## 3 Discussion

In the mCRC case presented here, ctDNA was monitored by liquid biopsy over time, allowing for rechallenge with anti-EGFR mAb treatment. The patient was initially diagnosed with *RAS* wild-type mCRC and was started on standard treatment with cetuximab + mFOLFOX6. The first ctDNA test was performed when second-line FOLFIRI + ramucirumab resulted in PD, and showed *RAS* mutation. The second ctDNA test was performed after fifth-line CAPOX + bevacizumab, but found no *RAS* mutation; thus, she was treated with CPT-11 + cetuximab, which resulted in PR with a progression-free response of 8 months. Furthermore, three ctDNA analyses after disease progression revealed positive *KRAS* and *NRAS* codon 61 mutation results, a rapid increase in *NRAS* codon 61 mutation levels over time, and no effect of anti-EGFR mAbs.

**Figure 4:**
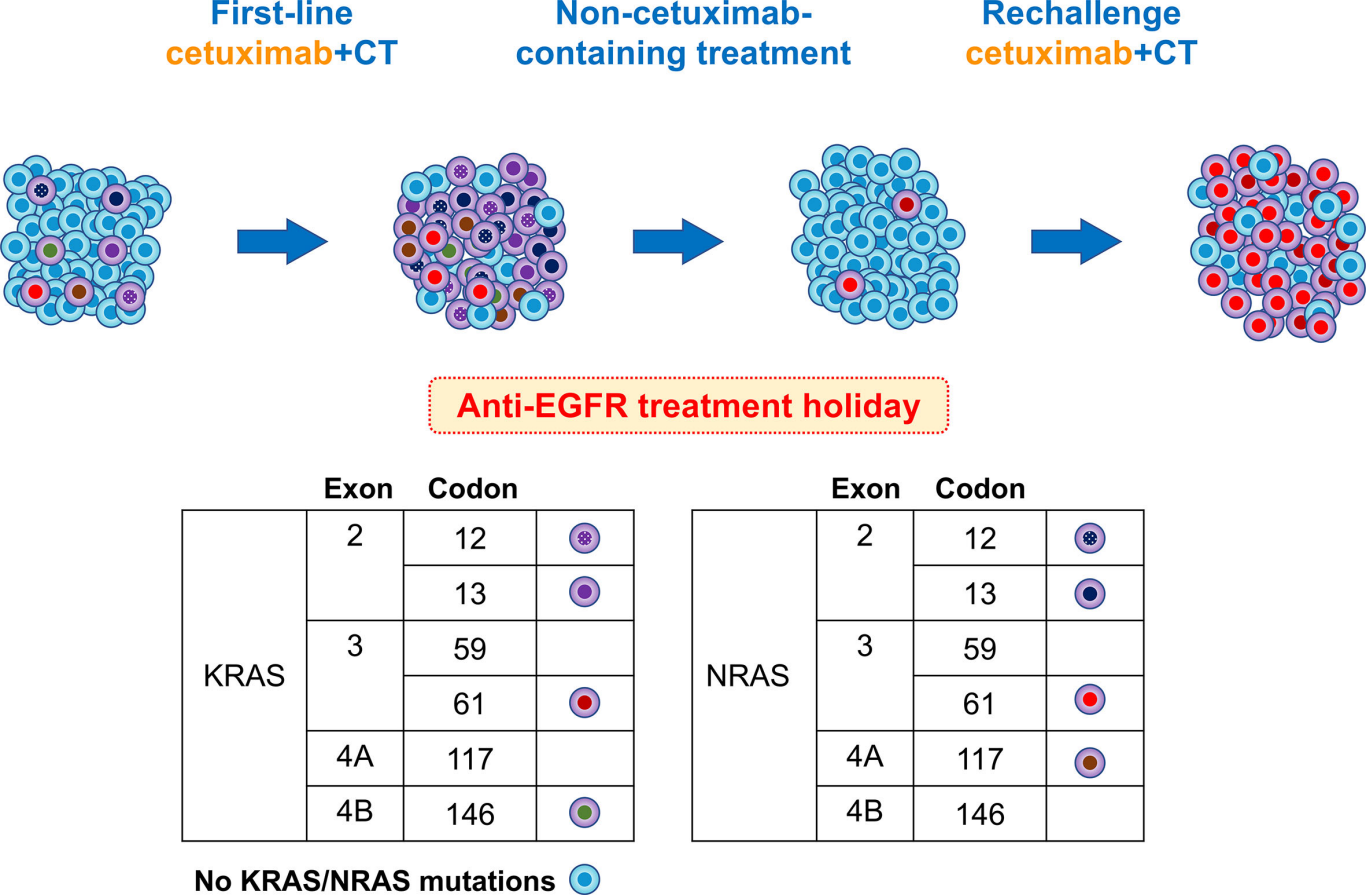
Schema of putative mutational changes in our patient treated with anti-EGFR. Newly detected *RAS* mutations in the plasma cfDNA of patients refractory to anti-EGFR therapy appear to be derived from a rare clone pre-existing in the primary tumor. Upon treatment with anti-EGFR antibodies, tumors experience a gain in new mutations. During progression, acquired resistance mutations were predominant, with a few remaining anti-EGFR-sensitive clones. After the discontinuation of anti-EGFR therapy, the number of RAS mutant subclones decreased or disappeared. In this case, *KRAS* codon 61 increased and *NRAS* codon 61 increased rapidly, and the patient again became resistant to anti-EGFR therapy.

Based on research findings reported to date, we present a hypothesis explaining the course of the *RAS* mutation in this case (**Figure 4**). Acquired resistance to anti-EGFR mAbs is associated with the emergence of *RAS* mutations (15, 16). *RAS* mutations are thought to be present at undetectable levels prior to the administration of anti-EGFR mAbs, and the number of *RAS*-mutant cells increases to detectable levels during administration of these mAbs (17). Thus, anti-EGFR mAbs exert selective pressure on heterogeneous tumors containing an undetectable *RAS*-mutant population, allowing *RAS*-mutant anti-EGFR-resistant cells to survive. Thus, *RAS* mutations may exist as subclonal mutations with low allele frequencies that are not detectable with the detection sensitivity of polymerase chain reaction-based methods, such as RASKET (16).

However, the proportion of mutated *RAS* alleles acquired during second-line therapy, without anti-EGFR mAbs, is expected to decrease below the detection limit (18). Russo et al. showed that treatment-stressed CRC cells can develop resistant clones, such as those with *RAS*/*EGFR*-ECD mutations, when EGFR is inhibited, by transiently reducing DNA repair pathways, such as mismatch repair and homologous recombination. This altered protein expression returns to baseline when the stress of the targeted therapy is removed, suggesting that this was a transient process (19).

In our case, a small number of RAS-mutant subclones were identified at the start of the third-line treatment (approximately 6 months after cetuximab withdrawal) when ctDNA was first measured. However, the RAS-mutant subclones disappeared at the 5th line of treatment (almost 11 months after cetuximab withdrawal). The half-life of *RAS* mutations after withdrawal of anti-EGFR mAbs was reported to be approximately 4.3 months (8), which is generally consistent with the course of our case. Thus, because *RAS*-mutant anti-EGFR mAb-resistant subclones do not necessarily disappear immediately after second-line therapy, subsequent tracking of *RAS* mutation dynamics can increase the likelihood of a successful rechallenge with an anti-EGFR-mAb.

In the present case, a mild increase in *KRAS* codon 61 and a rapid increase in *NRAS* codon 61 mutant allele frequency were observed after anti-EGFR-mAb rechallenge. Morelli et al. examined the frequency of newly detected acquired *KRAS* mutations in mCRC patients resistant to anti-EGFR mAbs and reported that *KRAS* codon 61 mutations were predominant, at 33%, making these mutations, along with codon 12 mutations, predictive biomarkers of anti-EGFR mAb-resistance (16). It has also been reported that *KRAS* codon 61 mutations are more frequently expressed as acquired-resistance mutations in individuals exposed to anti-EGFR therapy than in the general CRC population (20). However, *KRAS* codon 61 mutations have been shown to result in weak RAS-GTPase activity in transformation assays, resulting in a lower growth advantage than that of exon 2 mutations, which may expand only when tumors are subjected to therapeutic pressure with EGFR inhibitors (21).

Mutations in *NRAS* codon 61 are specifically associated with distant metastasis of thyroid cancer (22). In melanoma, *NRAS* codon 61 mutations have been reported to predominate over other oncogenic *NRAS* mutations and to promote melanoma formation, not because of differential involvement in downstream effector pathways, but because of the increased abundance of GTP-bound active forms (23). Based on these findings, *NRAS* codon 61 mutant clones may be more likely to gain a growth advantage in CRC. Based on these reports, *KRAS* codon 61 and *NRAS* codon 61 mutations may be predictive biomarkers of anti-EGFR mAb-resistance.

Acquired resistance to anti-EGFR-mAbs in CRC has been explained by a model in which new mutations are acquired in MAPK pathway members, such as KRAS/NRAS/EGFR. However, this is mainly based on clinical trials of anti-EGFR mAb monotherapy, and little is known about the mechanism of resistance to anti-EGFR-mAbs used in combination with cytotoxic chemotherapy. Given that the response rate to rechallenge with anti-EGFR therapy is approximately 30% (11) and that acquired resistance mutations have only been identified in 35–40% of patients (24), novel pathways leading to escape from anti-EGFR therapy may exist.

Recently, transient defects in mismatch repair (adaptive mutability model) (19, 25) and adaptive changes in the differentiation state and cell fate (epithelial–mesenchymal transition) (26) have been thought to contribute to acquired resistance to anti-EGFR therapy.

In addition, it has also been reported that, in the presence of cytotoxic chemotherapy combined with EGFR inhibitors, transcriptomic resistance mechanisms predominate over preexisting clonal growth (27). The short duration of response and rapid progression after cetuximab rechallenge in this case also suggested the presence of more complex acquired resistance mechanisms. Elucidation of these mechanisms will be important for an effective anti-EGFR rechallenge strategy.

There are limitations to ctDNA testing that should be considered. In general, detection limitations can prevent the identification of specific changes and can affect the accuracy of genetic profiling; however, plasma OncoBEAM has demonstrated sensitive detection ability with a mutant allele frequency of 0.02% (28). In addition, the overall percentage agreement between plasma-based and tissue-based *RAS* mutation testing was reported to be 84.6–90.4% (28–31). However, in some cases, the amount of ctDNA in mCRC patients, with a few lung metastases only or small lesion diameters, is low and caution should be taken in the interpretation of results when using OncoBEAM (31). Although ctDNA has advantages, such as non-invasiveness and continuous monitoring, these limitations underscore the need for careful interpretation. Research to address these limitations and to further improve the utility and accuracy of ctDNA testing is warranted.

## Data availability statement

The original data generated or analyzed during this study are included either in this article or in an additional file. Further inquiries can be directed to the corresponding authors.

## Ethics statement

Ethical review and approval was not required for the study on human participants in accordance with the local legislation and institutional requirements. Written informed consent was obtained from the individual for the publication of any potentially identifiable images or data included in this article.

## Author contributions

TS and YS conducted the literature searches and wrote the manuscript. TS treated the patient. HN, MH, KH, FT, and KF were part of the management team for the patient. KO, YK, MS, HM, and TT supervised the treatment and were involved in data analysis. All authors contributed to the article and approved the submitted version. TS and YS contributed equally.
