# Supplementary material for: Case Report: Longitudinal monitoring of clonal evolution by circulating tumor DNA for resistance to anti-EGFR antibody in a case of metastatic colorectal cancer
Source: Front Oncol. 2023 Jun 26;13:1203296. doi: 10.3389/fonc.2023.1203296 (PMC10332633; doi:10.3389/fonc.2023.1203296)
Supplement: Supplementary file 1 [file Table_1.docx]

Supplementary Material

Longitudinal monitoring of clonal evolution by circulating tumor DNA for resistance to anti-EGFR antibody in a case of metastatic colorectal cancer

Tamotsu Sagawa^1^, Yasushi Sato^2*^, Masahiro Hirakawa^1^, Kyoko Hamaguchi^1^, Fumito Tamura^1^, Hiroyuki Nagashima^1^, Koshi Fujikawa^1^, Koichi Okamoto^2^, Yutaka Kawano^2^, Masahiro Sogabe^2^, Hiroshi Miyamoto^2^, Tetsuji Takayama^2^

*** Correspondence:** Yasushi Sato
E-mail: [sato.yasushi@tokushima-u.ac.jp](mailto:sato.yasushi@tokushima-u.ac.jp)

# Supplementary Table S1.

# *KRAS* and *NRAS* mutations detected by OncoBEAM RAS CRC assay and Foundation-one liquid

|  |  |  | **RASKET-B** | **OncoBEAM ①** | **OncoBEAM ②** | **Foundation one Liquid** | **OncoBEAM ③** | **OncoBEAM ④** |
| --- | --- | --- | --- | --- | --- | --- | --- | --- |
|  |  |  | Apr-19 | Feb-21 | Sep-21 | May-22 | Sep-22 | Dec-22 |
| Gene | Exon | codon | Muted alleles (%) | | | | | |
| KRAS | 2 | 12 |  | 0.47383 |  |  |  |  |
|  |  | 13 |  | 0.11675 |  |  |  |  |
|  | 3 | 59 |  |  |  |  |  |  |
|  |  | 61 |  |  |  | Q61H (0.0073) | 0.04446 | 0.02960 |
|  | 4A | 117 |  |  |  |  |  |  |
|  | 4B | 146 |  | 0.12190 |  |  |  |  |
| NRAS | 2 | 12 |  | 0.51654 |  |  |  |  |
|  |  | 13 |  | 0.12688 |  |  |  |  |
|  | 3 | 59 |  |  |  |  |  |  |
|  |  | 61 |  | 0.04713 |  | Q61L (0.0214) | 1.92437 | 4.50708 |
|  | 4A | 117 |  | 0.10870 |  |  |  |  |
|  | 4B | 146 |  |  |  |  |  |  |

no mutation

mutation
